# Self-Reported Digital Literacy of the Pharmacy Workforce in North East Scotland

**DOI:** 10.3390/pharmacy3040182

**Published:** 2015-10-15

**Authors:** Katie MacLure, Derek Stewart

**Affiliations:** School of Pharmacy and Life Sciences, Robert Gordon University, Aberdeen AB10 7GJ, UK; E-Mail: d.stewart@rgu.ac.uk

**Keywords:** digital literacy, pharmacy workforce, pharmacy technology, ehealth, change management, Scotland

## Abstract

In their day-to-day practice, pharmacists, graduate (pre-registration) pharmacists, pharmacy technicians, dispensing assistants and medicines counter assistants use widely available office, retail and management information systems alongside dedicated pharmacy management and electronic health (ehealth) applications. The ability of pharmacy staff to use these applications at home and at work, also known as digital literacy or digital competence or e-skills, depends on personal experience and related education and training. The aim of this research was to gain insight into the self-reported digital literacy of the pharmacy workforce in the North East of Scotland. A purposive case sample survey was conducted across NHS Grampian in the NE of Scotland. Data collection was based on five items: sex, age band, role, pharmacy experience plus a final question about self-reported digital literacy. The study was conducted between August 2012 and March 2013 in 17 community and two hospital pharmacies. With few exceptions, pharmacy staff perceived their own digital literacy to be at a basic level. Secondary outcome measures of role, age, gender and work experience were not found to be clear determinants of digital literacy. Pharmacy staff need to be more digitally literate to harness technologies in pharmacy practice more effectively and efficiently.

## 1. Introduction

Pharmacy staff across all practice settings are increasingly reliant on information technology [1,2,3,4]. Pharmacists, graduate (pre-registration) pharmacists, pharmacy technicians, dispensing assistants and medicines counter assistants use widely available office, retail and management information systems alongside dedicated pharmacy management and electronic health (ehealth) applications in a range of community, hospital and other pharmacy settings. The abilities of pharmacy staff to use these applications at home and at work, also known as digital literacy or digital competence or e-skills, depends on personal experience and related education and training [5,6].

The British Computer Society defines digital literacy as, “being able to make use of technologies to participate in and contribute to modern social, cultural, political and economic life” [7]. A similar definition of digital literacy is adopted in the United States (U.S.), “the ability to use information and communication technologies to find, evaluate, create, and communicate information; it requires both technical and cognitive skills” [8]. Both definitions are grounded in historical and conceptual definitions of digital literacies [9]. United Nations Educational Scientific and Cultural Organisation (UNESCO) identifies digital literacy as both a “life skill” and “gate skill” because “it targets all areas of contemporary existence” [10]. The European Commission Information Society (ECIS) promotes and tracks citizens’ and member states’ digital engagement [11]. Similarly, the European Parliament promotes digital literacy for lifelong learning along with a recommendation for “better identification of occupational needs” [12].

A government commissioned report into digital literacy in Australia concluded that “both citizen and worker will need to be digitally literate for the digital economy to work effectively” while a report from New Zealand argues “that technology can change the nature of work faster than people can change their skills” [13,14]. In the United Kingdom (UK), a range of strategic principles, national competency frameworks for training, core skills and digital literacies for the general public, and recently more specific targets for the health sector, have been developed by government, advisory and healthcare related professional bodies [15,16,17].

Healthcare in the UK is politically devolved to its national parliaments. The Scottish Government’s policy driven National Health Service (NHS) is regionalised to 14 local Health Boards. Each Board manages the delivery of pharmacy services within hospitals and contracts community pharmacies to offer a range of core services most of which are technology based. In Scotland, community and hospital pharmacies are sited in remote and rural or urban settings with a diverse level of technology implemented, from the minimum required to facilitate electronic transfer of prescriptions (ETP) starting from a broadband networked single computer with linked printer and barcode reader through to advanced robotic medicines management systems [18].

The regulatory body for the pharmacy profession in the UK, which also sets standards for education and training, is the General Pharmaceutical Council (GPhC) [19]. Unlike the U.S., where the Accreditation Council for Pharmacy Education (ACPE) added health informatics in 2007, digital skills are not explicitly identified in pharmacy curricula in the UK [20].

However, the way ahead for the pharmacy workforce is a central issue in the most recent “Review of NHS Pharmaceutical Care of Patients in the Community in Scotland” [21]. It recommended, “developing and using the skills of the whole pharmacy team” to inform and support the “Prescription for Excellence” through person-centred, pharmaceutical care [22]. The main policy driver is the promotion of patient safety through upskilling and development of roles within the pharmacy workforce supported and facilitated by technology. The Scottish Government and NHS in Scotland envisage, “making more and better use of technology and facilities to increase access to services and improve efficiency,” also promising to ensure that everyone working within the integrated health and social care sector “is supported to make the best use of new technology” [23,24]. Policy and strategy drivers aim to change the role of pharmacy practice, upskilling the role of each member of the pharmacy team, within the integrated health and social care team, releasing the pharmacist for a more clinical, patient-facing role [25]. The policy driven intention is to support role development with technology therefore pharmacy needs a digitally literate workforce.

The aim of this research was to gain insight into the self-reported digital literacy of the pharmacy workforce in the North East (NE) of Scotland.

## 2. Experimental Section

### 2.1. Design

A purposive case sample survey was conducted across NHS Grampian in the NE of Scotland as part of a larger study.

### 2.2. Setting

NHS Grampian health board manages eight hospitals (two main in Aberdeen and Elgin) and 131 community pharmacies (51 in Aberdeen City, 53 in Aberdeenshire, 27 in Moray) that serve a population of over half a million across the North East of Scotland [26].

### 2.3. Sampling of Community and Hospital Pharmacies

Experts from the local health board and practicing academic pharmacists were engaged to assist with stratified purposive sampling taking into account the range of:

- urban, remote and rural geographical settings;
- technology infrastructures;
- pharmacy management systems implemented; and
- hospital pharmacy (community, major) and community pharmacy (small independent through to small, medium or large chain multiples; in the NHS Grampian area there are 23 small independents (single pharmacy), 67 small (1–4 pharmacies) to medium (5–25 pharmacies) chain multiples, and 41 national chain multiples) [26].

### 2.4. Recruitment of Pharmacy Owners and Managers

Professional networks of academic pharmacists based at Robert Gordon University (RGU), one of two centres offering graduate level pharmacy courses in Scotland, made the initial approach to local area network of pharmacy owners and managers. They outlined the proposed study and those who expressed an interest were emailed an information sheet and consent form by the research team. Management consent included facilitating contact with the wider pharmacy team.

### 2.5. Recruitment of Pharmacists, Pharmacy Technicians, Dispensing Assistants and Medicines Counter Assistants

Where distance allowed, the researcher arranged a preliminary site visit as a follow up to email contact. This served multiple purposes of introducing the researcher to potential participants, familiarising the researcher with the pharmacy location and layout and for distribution of information sheets and consent forms. It was emphasised that consent was both voluntary and individual. Where the preliminary visit was not possible a study pack was posted to the pharmacy.

### 2.6. Data Collection

The study was conducted between August 2012 and March 2013 in 17 community and two hospital pharmacies across the NHS Grampian area. Preliminary site vetting during sampling provided data on pharmacy setting (community/hospital), type (from small/medium/large single/independent/chain multiple), rural/urban, high/low technology and pharmacy management system. Categorisation as a “low tech” pharmacy was allocated where the minimum specification necessary to operate was implemented. For example, single or multiple PCs connected to a network server with secure N3 broadband connection, barcode scanner(s), label dispenser(s), printer(s) and, very often, a fax machine. If the pharmacy had robotic medicines management capability it was categorised as “high tech.”

During the consent process, participants provided five further items of data: sex, age band, role, pharmacy experience, plus answering a final question, “As a gauge of your current information technology experience which of the following would be the most appropriate challenge for you?”, followed by titles of six digital literacy courses listed in order of difficulty (Table 1), which formed the primary outcome measure for this study.

**Table 1:** Information Technology (IT) course titles descriptions listed in order of difficulty.

Course title		Description
**← Increasing level of difficulty**	Computing for the Terrified	If you are new to computing then this is the course for you. This short course gives you the opportunity to explore the basics of using a computer in a friendly and relaxed environment. Overcome your fears of using a computer and learn a new subject.
	Computing for the Quietly Confident	To provide students with a firm understanding of Microsoft Office applications. Learners should finish the course with a good grasp of word processing.
	Computing for the Courageous	Learners should finish the course with a more advanced grasp of word processing, basic spreadsheets, basic databases and basic presentations.
	European Computer Driving Licence (ECDL)	Attaining a European Computer Driving Licence is the best way to ensure you have all the necessary computing qualifications of any workplace. This course covers the first steps of using a computer—IT fundamentals, the internet, email and security.
	ECDL Advanced	Enables the learner to work more effectively with IT. This unit looks at using advanced tools to save time and effort when producing word processed documents, presentations and spread sheets.
	Computing Degree or Diploma	Course content includes: Computer Architecture, Computer Operating Systems 1; Computing: Planning; Computing: Graded Unit; Information Technology: Applications Software 1, Working within a Project Team.

Source of table content: local community learning advertisements placed by Aberdeenshire Council in conjunction with Aberdeen College (now NE Scotland College).

### 2.7. Data Analysis

The site data was collated and tabulated (Table 2) to show pharmacy types, description, rurality, number of pharmacy staff taking part, volume of dispensing, level of technology and pharmacy management system implemented. Participating pharmacies were described as small independent single or chain multiples (1–4 pharmacies) through large independent chain multiples (5–25 pharmacies), up to large chain multiples (>25 pharmacies) in the community sector plus two of the main hospitals in the area. Data gathered during the consent process were explored using descriptive statistics and presented in graphical form comparing: age, role, pharmacy experience and self-reported perception of digital literacy.

This study was approved by the Ethics Review Panel of Robert Gordon University School of Pharmacy and Life Sciences and deemed service evaluation by the Research & Development office of NHS Grampian.

**Table 2:** Overview of participating pharmacy demographics.

Case	Setting	chain multiple Type	Rural/Urban	P	PT	DA	MCA	No. of Rx per month	High tech/ Low tech	Pharmacy Management system
1	community	large, independent, chain multiple	R	1	1	0	4	8000	L	Cegidem
2	community	small, independent, chain multiple	U	1	0	1	3	5000	L	ProScript
3	community	small, independent, chain multiple	U	1	1	0	2	7000	L	ProScript
4	community	small, independent, chain multiple	R	1	1	0	1	4000	L	ProScript
5	community	small, independent, chain multiple	R	1	1	0	2	4500	L	Cegidem
6	community	small, independent, chain multiple	R	1	0	1	2	6500	L	Cegidem
7	community	small, independent, chain multiple	R	1	0	1	1	3000	L	Cegidem
8	community	small, independent, chain multiple	R	1	0	0	1	850	L	Cegidem
9	community	small, independent, chain multiple	R	0	0	0	1	1350 items	L	Cegidem
10	community	large, chain multiple	U	1	0	0	1	11,000	L	Nexphase
11	community	small, independent, chain multiple	U	3	2	1	2	Info withheld	H	Positive Solutions
12	community	small, independent, chain multiple	U	1	1	0	1	2500 items	L	ProScript
13	community	large, independent, chain multiple	R	1	1	0	2	3500	L	Cegidem
14	community	large, chain multiple	R	1	1	1	3	11,000	L	ProScript
15	community	small, independent, chain multiple	U	1	1	0	4	Info withheld	H	Positive Solutions
16	community	small, independent, chain multiple	U	3	0	2	2	8000	L	Cegidem
17	community	small, independent, chain multiple	U	2	2	2	2	Info withheld	H	Positive Solutions
18	hospital	medium	U	4	3	1	0	not available	L	JAC
19	hospital	large	U	1	4	5	0	> 800 items per day	H	JAC

Notes: small independent chain multiple (1–4 pharmacies); large independent chain multiple (5–25 pharmacies); large chain multiple (> 25 pharmacies); P = Pharmacist; PT = Pharmacy Technician; DA = Dispensing Assistant; MCA = Medicines Counter Assistant; Rx = prescription.

## 3. Results and Discussion

### 3.1. Types of Pharmacy and Settings

Two of the community pharmacies that had expressed an initial interest in taking part in the study withdrew before the consent process due to staff illness and holiday commitments. No potential participants withheld consent or subsequently withdrew from the study. The figures given reflect the number in each role available and willing to participate on the arranged day and time which is not necessarily the number employed at that pharmacy.

Although prescriptions and item quantities dispensed are not directly comparable, it provided an impression of dispensing volume per pharmacy. These ranged from approximately 850 prescriptions per month in a small, rural community pharmacy to over 800 items per day in a large, hospital pharmacy dispensary. Three community pharmacies withheld dispensing volume information citing commercial confidentiality reasons and the information was not available from one of the hospital.

### 3.2. Hardware and Software Implemented

Fourteen of the community pharmacies and one hospital pharmacy were categorised as “low tech” with three community and one hospital pharmacy deemed “high tech.” Neither hospital had introduced HEPMA (hospital electronic prescribing and medicines administration). A range of commercially available pharmacy management software applications were implemented. Two ran the specialist hospital pharmacy software, JAC, while the community pharmacy systems included Cegidem (*n* = 8), Positive Solutions (*n* = 3), ProScript (*n* = 5) and Nexphase (*n* = 1).

### 3.3. Participant Demographics

There were 94 participants from the 19 pharmacies including:

- 24 pharmacists, two of whom were locums;
- 2 pre-registration pharmacy graduates;
- 19 pharmacy technicians;
- 15 dispensing assistants; and
- 34 medicines counter assistants.

**Figure 1:**
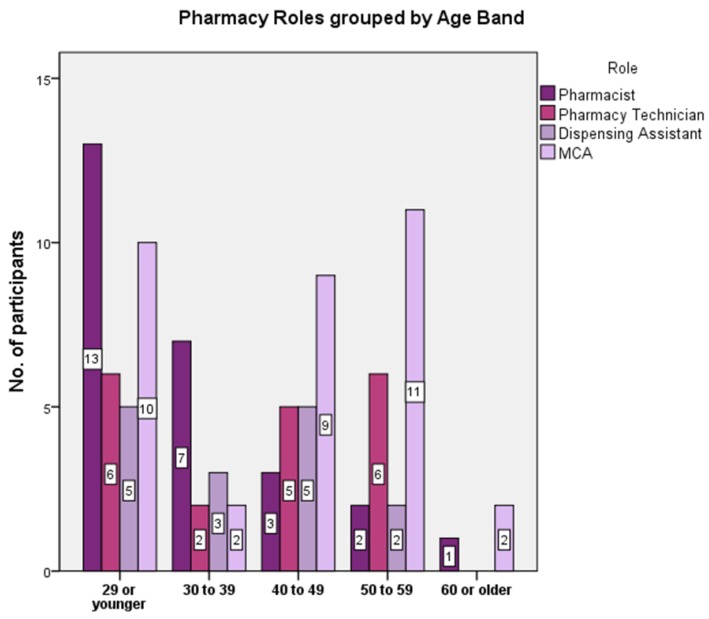
Participant roles grouped by age band.

Of the 13 male participants, ten were pharmacists, one was a dispensing assistant and two were medicines counter assistants. While half the pharmacists were aged 29 or younger, other pharmacy staff groups featured a broader age range (Figure 1). The participants’ experience working in pharmacy ranged from an MCA with one-month experience to 35 years, also an MCA. The sample population showed similarities in the age range, sex and role to the national demographics [27].

**Figure 2:**
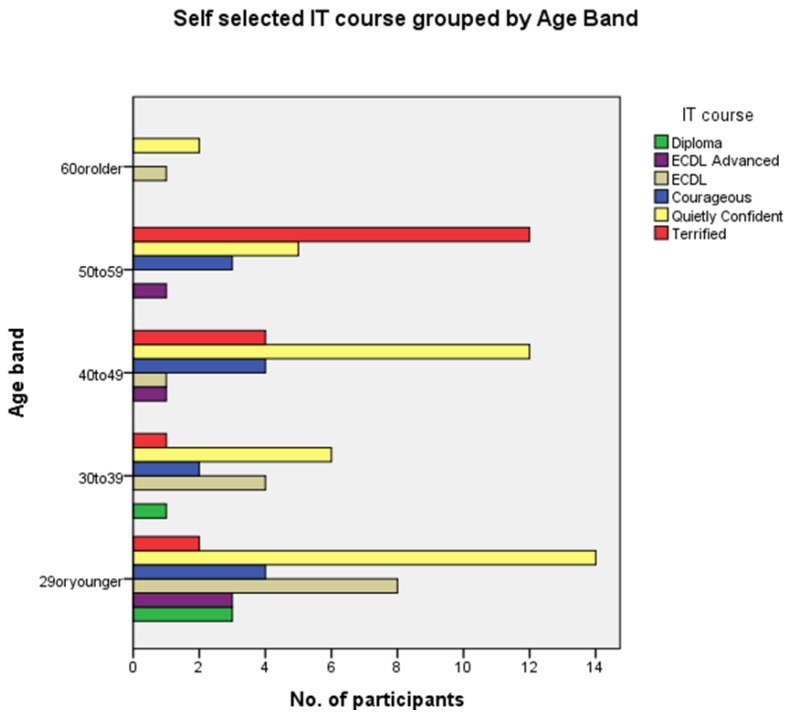
Self-selected IT course by age band.

As previously described, participants self-selected from six IT course titles listed in order of difficulty (Table 1) as a self-reported measure of their digital literacy. The most frequently self-selected IT course across all pharmacy roles (Figure 2) was “Computing for the Quietly Confident” (*n* = 39) followed by “Computing for the Terrified” (*n* = 19). These two least difficult courses together accounted for the selections of nearly two-thirds of participants. The remainder selected “European Computer Driving Licence” (ECDL; *n* = 14), “Computing for the Courageous” (*n* = 13), “ECDL Advanced” (*n* = 5) or “Degree or Diploma” (*n* = 4).

**Figure 3:**
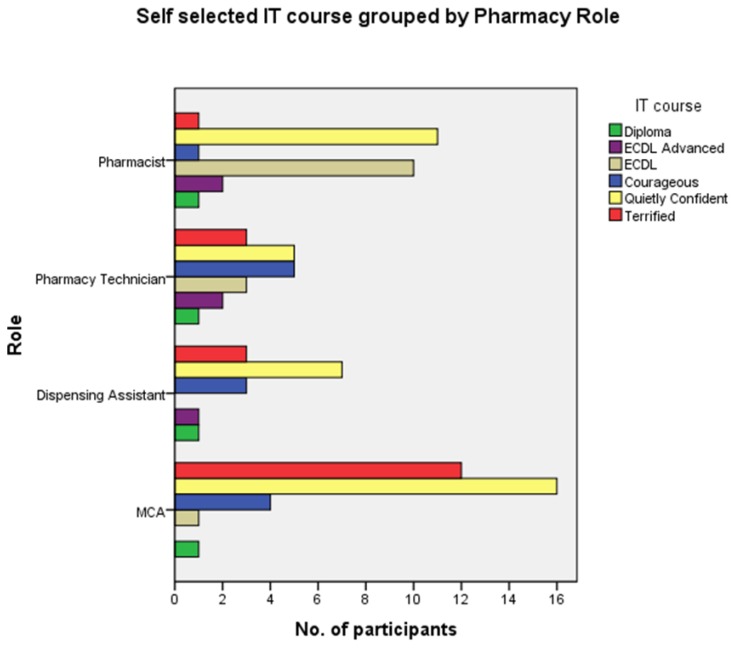
Self-selected IT course by pharmacy role.

Similarly, grouping self-selected IT course by age band, showed “Computing for the Quietly Confident” as the most frequently selected in all age bands except “50 to 59” where “Computing for the Terrified” was the predominant option selected (Figure 2). “Computing for the Terrified” featured as a choice for all age bands except the “60 or older.” Although one third of pharmacists (*n* = 8) in the “29 or younger” age band self-selected “ECDL” as their appropriate IT challenge, the predominance of the lower level courses, indicative of basic levels of digital literacy, was clear across all roles and age bands (Figure 3).

### 3.4. Key Findings

With few exceptions, pharmacy staff in the NHS Grampian area work with minimum levels of technology and perceive their own digital literacy to be at a basic level. Role, age, gender and work experience were not clear determinants of digital literacy. Pharmacy staff need to be trained, supported and mentored to harness pharmacy technologies effectively and efficiently in support of pharmacy practice development.

### 3.5. Strengths and Weaknesses

To the best of our knowledge, this is the first study to focus on the whole pharmacy team and their digital literacy. The generalisability of the study is strengthened by its stratification of pharmacy staff from all roles, age bands and with varying lengths of pharmacy experience. Sampling had facilitated access to a breadth of pharmacy type, setting, level of technology and pharmacy management system implementation. However, the study is limited by the inherent bias of self-reporting, its geographical focus on a relatively small sample, from one local health board area in the North East of Scotland.

### 3.6. Interpretation in Context with Existing Literature

Greenhalgh *et al.* explored the diffusion of innovation in health service organisations from several theoretical perspectives including Rogers “Adopter Categories” and “Attributes of Innovation” to account for the complexity of combining socio and technical systems [28,29]. This study identifies socio-technical and political competing forces at play which Lewin described in his Force Field theory as where, “an issue is held in balance by the interaction of two opposing sets of forces—those seeking to promote change (driving forces) and those attempting to maintain the status quo (restraining forces)” [30].

### 3.7. Driving Forces for Technology in Pharmacy

Clear driving forces for technology in pharmacy to support healthcare have been evidenced nationally by the Scottish Government through the:

- “eHealth Strategy” [2];
- “Better eHealth: Better Care—Citizen eHealth Survey” [31];
- “2020 Vision for Quality” [32];
- “2020 Route Map” [33];
- “Prescription for Excellence” [22]; and
- locally, by NHS Grampian [26]

Technology in pharmacy has been supported jointly by RPS & Royal College of General Practitioners, by the Royal Pharmaceutical Society, the GPhC with further backing from the “Review of Pharmaceutical Care of Patients in the Community”, endorsed by Community Pharmacy Scotland [15,16,19,21,34].

Another driving force is the educational support designed for the healthcare workforce to provide the digital literacy skills needed to use technology in pharmacy through the “2020 Workforce Vision”, NHS Knowledge & Skills Framework and British Computer Society “Preparing the NHS for an information revolution” to support upskilling of the workforce [23,35,36].

Societal healthcare needs and technological advances have driven the organisational adoption decision in favour of technology but this study demonstrates the need to upskill all members of the pharmacy team to effectively and efficiently harness change.

### 3.8. Restraining Forces for Technology in Pharmacy

If policy is the driver bringing technology into pharmacy practice, the main restraining forces are the pharmacy team, its leadership and the individuals within the pharmacy team. Findings depict a workforce who self-reported their digital literacy levels as basic. Although a small sample (*n* = 94), the evidence seems to counter the commonly held perception that younger, professional people are more digitally literate. Although there were notable exceptions, most pharmacies had the minimum level of technology implemented with unaddressed usability issues acting as barriers, or additional restraining forces. Changing roles may also lead to the pharmacist spending less time with the pharmacy team depriving the team of the cascading of knowledge. The decisions of individuals to adopt technology may be influenced by their use of technology outside work but Adair and Lewin would also argue that leadership and group acceptance of technology are influential in the change management needed to counter the restraining forces [37,38].

### 3.9. Conditions for Change

In “Human Relations in Curriculum Change,” Lewin outlined the conditions for change, the tensions at play in his Force Field theory and combinations of educational and organisational measures to “change the strength of opposing forces” [30,38]. Lewin considered the power of “social habits and group standards,” where there is resistance to change, to be amenable to a three step process of “unfreeze, change, refreeze”. Pharmacy worldwide employs a capable and skilled workforce. Providing the opportunity and motivation for behavioural change will be part of the on-going challenge [39].

### 3.10. Further Work

To the best of our knowledge, this is the only study to consider the digital literacy of the whole pharmacy team in both community and hospital settings. An observational case study is underway which will build on these findings. National surveys of digital literacy amongst not just the pharmacy workforce but also the broader healthcare workforce would provide a benchmark for evaluation of educational interventions and inform curricula design. Evaluation of progress towards implementation of policy around adoption of technology to support role development across the pharmacy team is also indicated.

## 4. Conclusions

Although this was a local study, the findings hold implications at the national level. Evidence from the findings suggests there is a policy driven intention to support changing roles in pharmacy by increasing and improving the provision of technology and providing the associated education and training of all pharmacy staff. With few exceptions, pharmacy staff in the NHS Grampian area work with minimum levels of technology and self-report their own digital literacy levels as basic. This tends to indicate organisational and social factors may act as restraining factors against the driving forces for technology in pharmacy and associated digital literacy training. Aspirational national and international policies have been shown to need local solutions for adoption to be successful [40]. Grudin’s law warns that, “when those who benefit are not those who do the work, the system is doomed to fail” [41]. Engaging and supporting the pharmacy workforce to improve their digital literacy is key to embracing greater access to technology in pharmacy practice and providing the opportunity to “unfreeze, change, refreeze” will be key to its success.

### Key Messages

- With few exceptions, pharmacy staff in the NHS Grampian area work with minimum levels of technology and perceive their own digital literacy to be at a basic level.
- Role, age, gender and work experience were not clear determinants of digital literacy.
- Pharmacy staff need to be trained to harness pharmacy technologies effectively and efficiently in support of their role development.
